# Silver nanoparticles synthesized from the seaweed *Sargassum polycystum* and screening for their biological potential

**DOI:** 10.1038/s41598-022-18379-2

**Published:** 2022-08-30

**Authors:** Rajasekar Thiurunavukkarau, Sabarika Shanmugam, Kumaran Subramanian, Priyadarshini Pandi, Gangatharan Muralitharan, Maryshamya Arokiarajan, Karthika Kasinathan, Anbarasu Sivaraj, Revathy Kalyanasundaram, Suliman Yousef AlOmar, Velmurugan Shanmugam

**Affiliations:** 1 Centre for Drug Discovery and Development, Col. Dr. Jeppiaar Research Park, Sathyabama Institute of Science and Technology Jeppiaar Nagar, Rajiv Gandhi Road, Chennai, 600 119 India; 2 Departmentof Microbiology, Centre of Excellence in Life Sciences, Bharathidasan University, Tiruchirappalli, 620 024 Tami Nadu India; 3 Department of Biotechnology, Mohamed Sathak College of Arts and Science, Chennai, Tamil Nadu India; 4 Department of Zoology, College of Science, Kind Saud University, Riyadh, 11451 Kingdom of Saudi Arabia; 5 Madda Walabu University, Robe, Ethiopia

**Keywords:** Drug discovery, Microbiology, Nanoscience and technology

## Abstract

World-wide antimicrobial resistant is biggest threat in global health. It requires the urgent need of multisectoral action for the scientific community to achieve the sustainable development Goals. Due to their antimicrobial properties, silver nanoparticles are potential activates to pathogens, which explains their potential for multiple applications in various fields. In the present studies, we evaluate the antimicrobial properties of a *Sargassum polycystum* algal extract, an unrivaled green synthetic method for producing -defined shaped seaweed silver nanoparticles. To confirm their structure and size, some characterization techniques are used, such as Absorption spectrophotometer (UV–VIS), Fourier transforms infrared spectroscopy (FTIR), Scanning electron Microscope (SEM), Transmission electron microscopy (TEM) and X-Ray diffraction (XRD). Evaluate the antibacterial and anti-mycobacterial activity using silver nanoparticles. The toxicity study of this silver nanoparticle has been done with the help of zebrafish larva. The biological nanoparticle having good antimicrobial activity against *Staphylococcus aureus*, *Micrococcus luteus*, *Pseudomonas fluorescens* and *Candida albicans* and also it shows potent activity against MTB H37Rv, SHRE sensitive MTB Rifampicin resistant MTB around 98%. Seaweed nanoparticles had lower toxicity for the survival of the fish larvae. In comparison, other dosages will arrest the cell cycle and leads to death. The present finding revealed that these seaweeds nanoparticles have potential anti-mycobacterial activity against pathogens at low concentrations. This makes them a potent source of antibacterial and anti-TB agents

## Introduction

Tuberculosis (TB) is an infectious disease and is one of the top 10 causes of the death globally^1^. *Mycobacterium tuberculosis* (MTB) is a causative agent for TB which affects lungs and also other parts of body except hair and nail. TB transmitted to other individuals through the aerosols expelled by infected people when they cough and the symptoms are cough, fever, night sweat and weight loss^2^. In many Middle- and low-income countries, it would be a main cause of mortality and morbidity. Multi drug resistant and extensively resistant TB are the major challenge in effective control of the disease in many regions^3^. TB treatment was usually take a prolonged time of the treatment of the TB patients with Anti-TB drugs^4^. The emergence of multidrug resistance (MDR) bacteria has prompted the development of new antibacterial medicines.

Silver Nanoparticles have been broad-spectrum, strong antibacterial activity against a variety of pathogens due to their small size and wide surface area. Low quantities of AgNPs can effectively destroy bacterial and viral pathogens such as *E. coli*, *Staphylococcus aureus*, *Klebsiella pneumoniae*, *Candida albicans*, *Aspergillus niger*, HIV, and the *hepatitis B virus *(HBV)^5^. Hence, we are searching for a novel well activated compound with lesser side effects. The synthesis of eco-friendly NPs is urgently needed to replace toxic chemicals in various fields. Biosynthesized Silver nanoparticles arecost-effective and eco-friendly biocompatible agent that possess the potential for biomedical and pharmaceutical applications^6^. Microorganisms such as mushroom, bacteria and algae, as well as plant extracts, contain enzymes, alkaloids, terpenoids, and phenolic compounds that can be used as stabilizers and capping agents during the biological synthesis of NPs^7^.

Nanoparticles has antimicrobial mechanism of action is said to follow one of three models: development of oxidative stress, metal ion release, or non-oxidative processes^8^. These three different mechanisms can all take place at the same time. According to certain research, Ag Nanoparticles cause the bacterial membrane's surface electric charge to be neutralised and alter its permeability, which ultimately results in bacterial death^9^. Additionally, the production of reactive oxygen species (ROS) impairs the antioxidant defence system and breaks down the cell membrane mechanically. The main mechanisms behind the antibacterial actions of NPs, according to current research, are as follows: (1) bacterial cell membrane disruption; (2) production of ROS; (3) cell membrane penetration; and (4) development of intracellular antimicrobial effects, including interactions.

Due to their relatively moderate side effects, marine resources are currently being intensively examined for antibacterial and anticancer medication candidates^8^. Marine algae, such as Chlorophyta (green), Phaeophyta (brown), and Rhodophyta (red), are considered highly potent renewable living marine resources, and their production of NPs has piqued interest. Polysaccharides, proteins, carbohydrates, vitamins, pigments, enzymes, and secondary metabolites, among other organic substances found in algae, provide additional potential for their function in the production of Silver nanoparticles by acting as natural reducing agents^11^.

Seaweeds are mostly used in industrial purpose, but they not yet extensively globally, seaweeds are exploited as the raw material for various industrial products, but they are not yet extensively imposing for nanoparticle biosynthesis. In this there are lesser number of studies are available on the synthesis of silver nanoparticle by seaweed and their antibacterial and anti-proliferative^12^, antifungal^13^ and anticancer^14^. Many seaweeds have potentiality to work against various disease rather some have been tested for clinical trial and next for medicine preparation. Some seaweed is known against human normal and multidrug resistant pathogens, to find out the best potent seaweed for synthesis of Silver Nanoparticle and its potential against the human pathogens. There are more than 841 seaweeds have been reported from the Indian coast having good potential over human pathogen. Chemical methods have adverse side effect on human and environment hence, we are going for a biological synthesis of silver nanoparticle.

In this paper we evaluated *Sargassum polycystum* seaweeds aqueous extract for the preparation of silver nanoparticle through green synthesis method. The spectroscopic techniques that have been used for the characterized the silver nanoparticles, such as absorption spectrophotometer (UV–VIS), Fourier transforms infrared spectroscopy (FTIR), scanning electron Microscope (SEM), transmission electron microscopy (TEM) X-Ray diffraction (XRD) and Dynamic light scattering (DLS). The effect of nanoparticle was analyzed and performed antimicrobial and anti-TB activity against various microbial pathogens. The toxicity of the synthesized silver nanoparticle was monitored through zebra fish larva.

## Material and methods

### Ethical statement

The experiment was conducted in line with the norms and regulations of the Institutional Ethical Committee (IEC) of Sathyabama Institute of Science and Technology (1793/PO/REBI/S/2014/CPCSEA) Chennai. All animal experimental protocols were approved by the Sathyabama Institute of Science and Technology's Institutional Animal Ethical Committee (IAEC). The ARRIVE guidelines (https://arriveguidelines.org/arrive-guidelines/experimental-procedures) were followed throughout the project.

### Microbial strains

*Escherichia coli* (MTCC1687), *Bacillus subtilis* (MTCC441), *Klebsiella pnemoniae* (MTCC4030), *Staphylococcus epidermidis* (MTCC435), *Vibrio cholera* (MTCC0139), *Pseudomonas fluorescens *(MTCC664), *Micrococcus luteus* (MTCC4821), *Staphylococcus aureus* (MTCC 96), *Serratia marcescens* (MTCC86), were used to test antimicrobial activity. *M. tuberculosis* H37Rv, SHRE sensitive *M. tuberculosis* and Rifampicin resistant *M. tuberculosis* were used for anti-mycobacterial activity. All studies were conducted at Centre for drug discovery and development, Sathyabama Institute of Science and Technology (Deemed to be University), Chennai.

### Collection of samples

Seaweeds (*Sargassum polycystum*, *Acanthophora spicifera*, and *Ulva fasciata)*are collected during the June–Aug 2018 at low tide and 5–6 mm dept from the coastal regions of Mandapam (Southeast coast of India). Samples are identified by Seaweed Expert Dr. P. Anantharaman Dean & Professor CAS in Marine Biology Faculty of Marine Sciences, Annamalai University Parangipettai-608 502. Marine seaweeds were characterized using Common Seaweeds and Seagrasses of India, Herbarium Vol.1 authenticated by Central Marine Fisheries Research Institute, (Indian Council of Agricultural Research), Kochi-682 018, Kerala, lndia. Seaweed samples were taken directly and quickly washed in seawater to remove any foreign particles, sands, or epiphytes. It was then placed in an ice box and carried to the laboratory, where it was properly washed with running tap water and then distilled water to eliminate any leftover adhesive particles, salt, or dust. The seaweeds were then spread out on blotting paper to absorb any excess moisture. It was dried in a dark room for 3–4 days. Using a mixer grinder, the dried components were ground to a fine powder.

### Preparation of seaweed extract

1 g of dried seaweed powder was placed in 250 ml conical flask and dissolved with 100 ml of distilled water. The mixture was boiled in a water bath for 20 min at 65 °C. The crude extract was filtered through Whatman Grade 1 Qualitative Filter Paper. Then the filtered extract was centrifuged for 10 min at 1800 rpm. Further the supernatant was stored at 4 °C and used for further studies.

### Biological synthesis of silver nanoparticles

10 mL of seaweed aqueous extract was added to 90 mL (0.1 mM)AgNO_3_ solutions and stored at room temperature for 24–48 h in the dark. After Incubation that samples were purified by centrifugation for 13,000 rpm for 20 min and remove the supernatant and dispersion of silver nanoparticles pellet in HPLC grade distilled water to remove the unbound particles for the future characterization of silver nanoparticles.

### Characterization of the silvernanoparticles

Formation of silver nanoparticles was confirmed by Ultraviolet–visible spectral analysis. The absorbance spectra were recorded using Ultraviolet–visible spectroscopy (UV-1800 Shimadzu UV spectrophotometer) at a wavelength of 200–800 nm. Fourier transforms infrared spectroscopy (Shimadzu, Japan) to find out the feasible functional group in the bioactive compounds of the seaweeds extract. Transmission electron microscopy was used to examine the size and form of the Ag NPs (TEM). The morphological properties of the silver nanoparticles were studied using a scanning electron microscope (SEM), and the crystalline nature, quality, and crystallographic determination of the silver nanoparticles were determined using an X-ray diffractometer. To calculate the polydispersity index (PDI) of nanoparticles, silver nanoparticles were examined in dynamic light scattering (DLS). Dynamic light scattering (DLS) (Zetasizer NanoR Model S90; Malvern Instruments, UK) was used to measure the dispersion, homogeneity, and nanoemulsion size in order to calculate the polydispersity index (PDI) of nanoemulsions. Three copies of each measurement calculation were made. A laser with a wavelength of 780 nm and a scattering angle of 90° was used to conduct measurements using dynamic light scattering (DLS) in the range of 0.1–1000 m at 25 °C.

### Antibacterial activity of silver nanoparticles

The antimicrobial activity of synthesized silver nanoparticle was tested against various pathogenic bacteria such as, *Escherichia coli*, *Klebsiella pneumonia*, *Bacillus subtilis*, *Micrococcus luteus*, *Staphylococcus aureus*, *Staphylococcus epidermidis*, *Vibrio cholera*, *Serratia marcescens* by agar well diffusion method. Each bacterial culture was grown in nutrient broth medium for 18 h at 37 °C. Then, each grown cultures were swabbed on nutrient agar medium and the well were cut about 5 mm using cork borer. Each well was added with 50 µl of synthesized silver nanoparticles and all plates were incubated at 37 °C for 18 h. After incubation, the plates were observing and the inhibited clear zone was measured and calculated^15^. The minimal inhibitory concentration (MIC) of the green synthesized silver nanoparticle was observed against each pathogenic bacteria using different concentration of nanoparticles. Based on the silver nanoparticle screening results, the bacterial pathogens such as, *Serratiamarcescens*, *Bacillus cereus*, *Escherichia coli*, *Micrococcus luteus* are used for further study. Among the bacterial strain the MIC wasobserved ranging from 16 to 256 μg/ml.

### Antimycobacterial activity

Luciferase reporter phage (LRP) assay was used to screen for anti-TB activity of synthesised silver nanoparticles mediated by seaweed. About 100 µL of *M. tuberculosis* H37Rv cell suspension (McFarland Unit 2) was added into 350 µL of sterile middlebrook 7H9 broth (Himedia) containing 50 µL of 32 synthesised silver nanoparticles at concentration of 100 µg/mL. Then it was incubated at 37 °C for 72 h. About 50 µL of mycobacteriophage (phAE202) and 40 µL of 0.1 M CaCl_2_ was added and incubated. After4 h at 37 °C, 100 µL of D-luciferin substrate was added to cell-phage mixture and relative light unit (RLU) was measured immediately at 10 s integration time in luminometer (Lumat 9508, Berthold, Germany). The percentage of RLU reduction was calculated by following formula:

$${\text{Control RLU }}{-}{\text{ test RLU}}/{\text{control RLU }} \times { 1}00.$$

Nanoparticles showed more than 50% RLU reduction were considered as having anti-mycobacterial activity. The same experiment was followed for sensitive *M. tuberculosis* and Rifampicin *M. tuberculosis* strains^16^.

### Toxicity evaluation of silver nanoparticles against zebra fish

Zebra fish embryos were purchased from the zebra fish aquarium in Kanchipuram district. For toxicity studies, 15 healthy post hatched zebra fish were transferred to the wells of a 24-well plate along with 1 ml of embryo water (60 mg of sea salt/l of ultrapure water). Different concentrations of silver nanoparticle (5, 10, 25, 50 and100 μg ml^−1^) were added to the wells and incubated for 72 h at 28.5 °C. Tests were performed in duplicate and repeated thrice (60 embryos per concentration). Mortality of the zebra fish was noted after 24, 48 and 72 h. The embryos that appeared opaque and white in colour. The dead embryos were degraded without any distinguished characteristic, whereas the structures of intact embryos were more visible distinguished characteristic by 48 h which allowed a clear distinction between the dead and alive. The mortality rate was observed and calculated. The embryos were photographed using an inverted phase contrast microscope (Olympus ckx41).

## Results

### Synthesis of silver nanoparticles

In this study we have performed green synthesis of silver nanoparticles. The three different seaweeds extract were mixed with silver nitrate and incubated at room temperature. The colour changes were monitored and observed. The experiments were performed with triplicate reaction. Formation Yellowish-brown colour confirmed the nanoparticle formation. There was no colour change in Control silver nitrate solution.

### Analysis of the silver nanoparticles using UV-spectrophotometer

**Figure 1:**
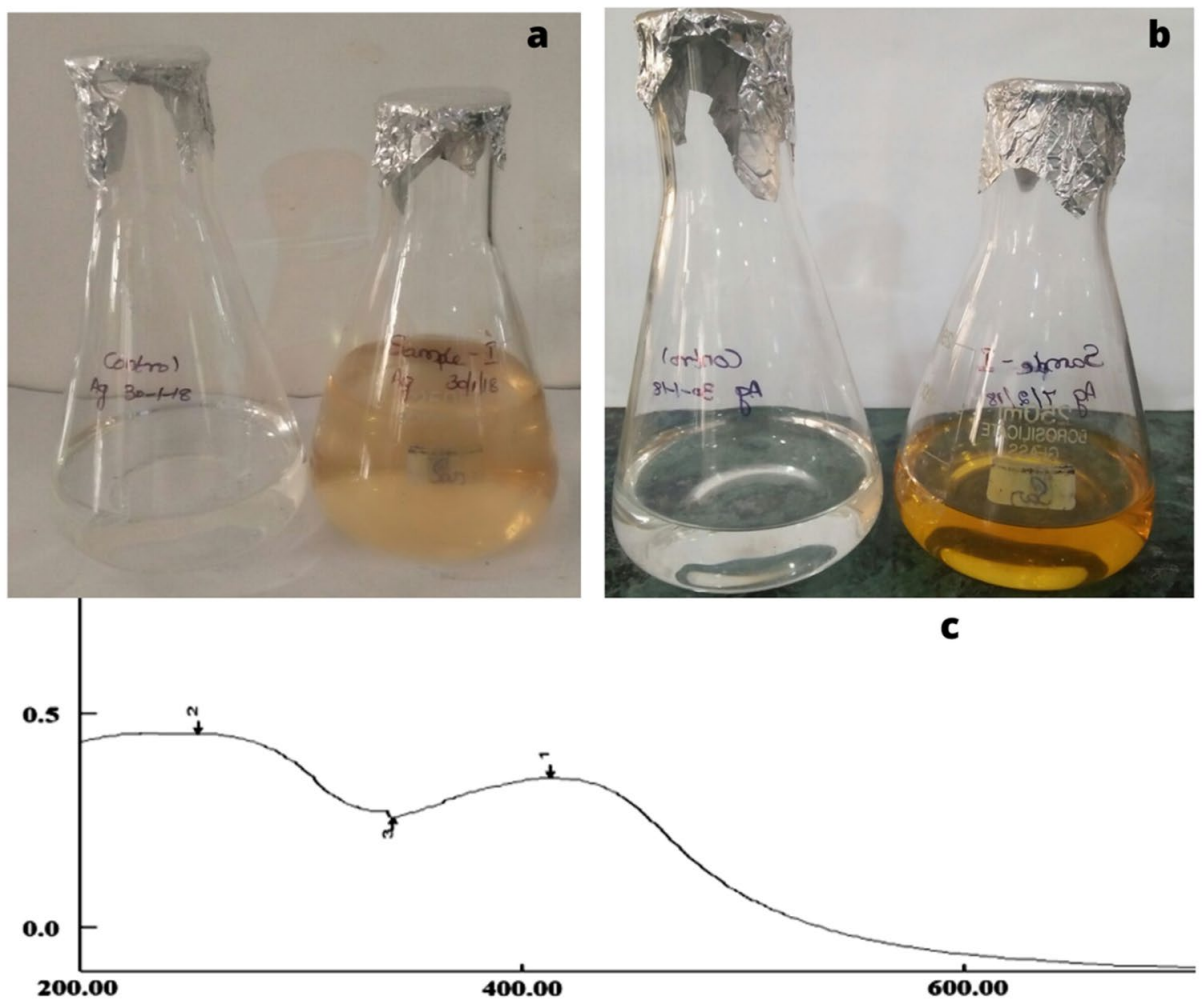
Biosynthesis of silver nanoparticles. (**a**) Before incubation. (**b**) After 48 h of Incubation and (**c**) UV–visible spectrophotometry of silver nanoparticles synthesized from extract of *Sargassum polycystum.*

**Figure 2:**
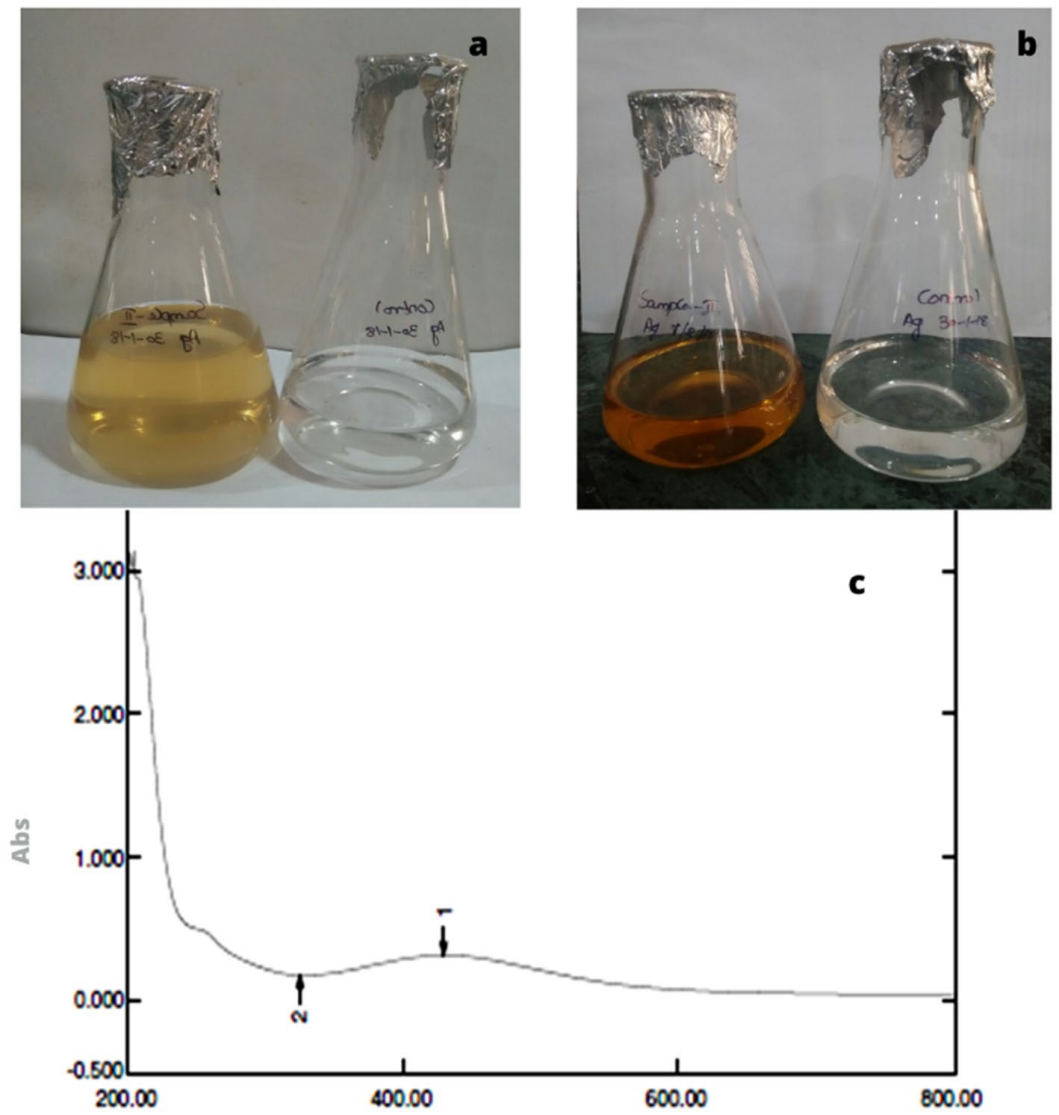
Biosynthesis of silver nanoparticles. (**a**) Before incubation. (**b**) After 48 h of Incubation and (**c**) UV–visible spectrophotometer of silver nanoparticles synthesized from extract of *Acanthophora spicifera.*

**Figure 3:**
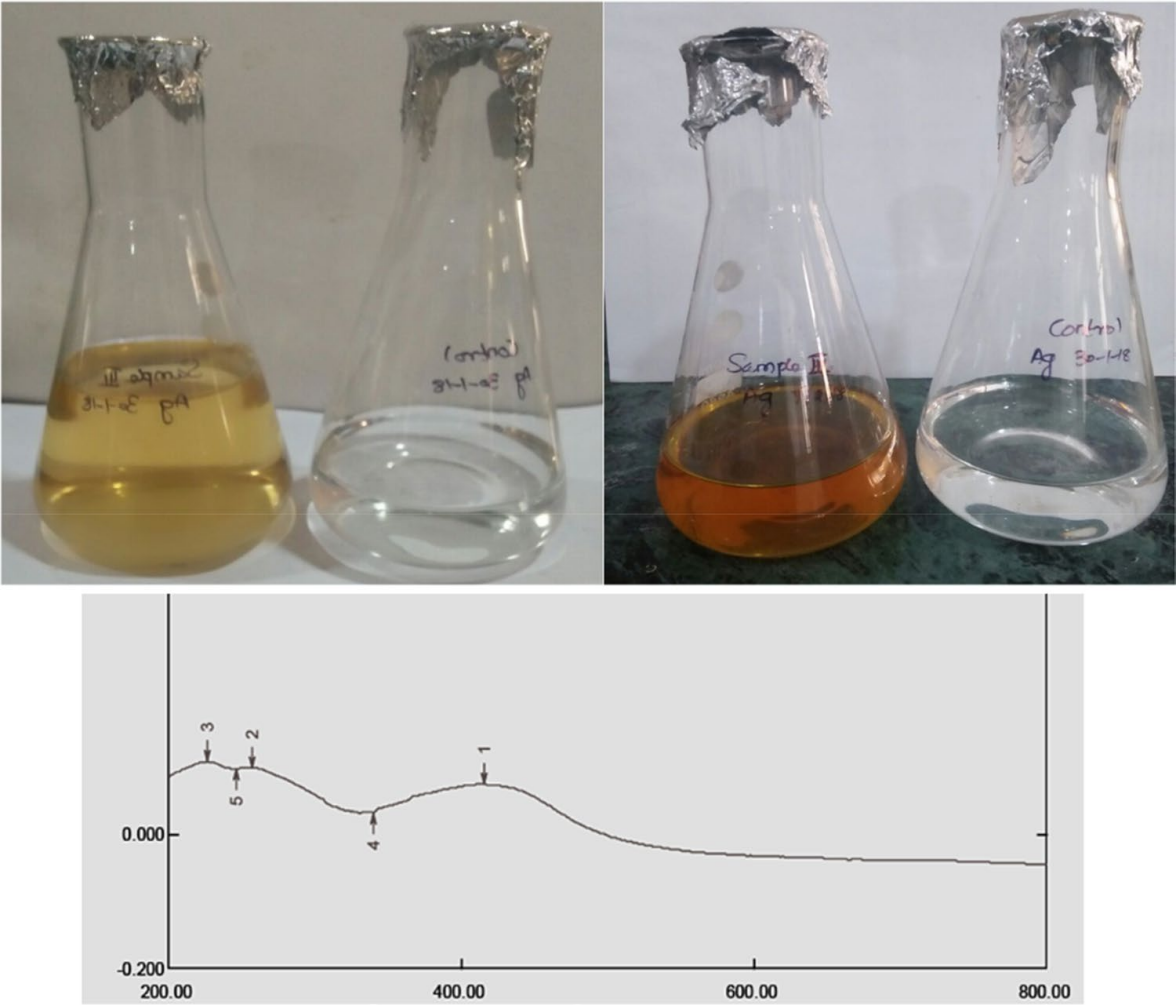
Biosynthesis of silver nanoparticles. (**a**) Before incubation. (**b**) After 48 h of Incubation and (**c**) UV–visible spectrophotometer of silver nanoparticles synthesized from extract of *Ulva fasciata.*

The synthesized silver nanoparticles were confirmed by using UV-spectrophotometer. Further confirmation of the formation of the silver nanoparticles was determined by UV–vis spectrophotometer. UV–Vis spectrum was showed in Figs. 1, 2 and 3. The significant observance of *Sargassum polycystum*, *Acanthophora spicifera*, and *Sargassum wightii* were showed at 422, 429 and 411 nm respectively. This absorption band called as surface plasmon resonance (SRP).

### Fourier transforms infrared (FT-IR) spectroscopy analysis

**Figure 4:**
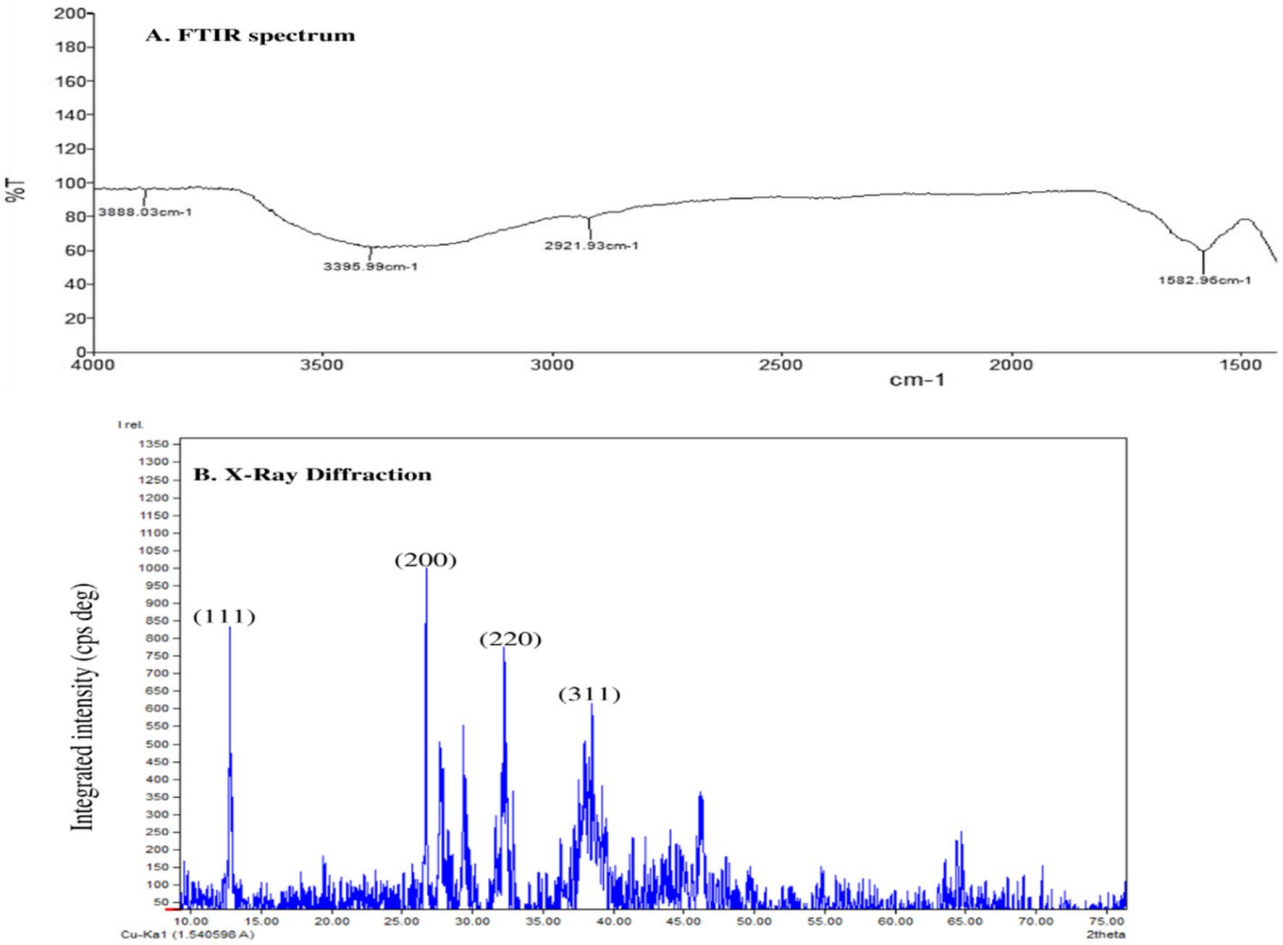
(**A**) Analysis of Silver nanoparticles using FTIR spectrum of Sargassum polycystum. (**B**) Analysis of Silver nanoparticles using X-Ray Diffraction of Sargassum polycystum.

FT-IR spectroscopic analysis of *Sargassum polycystum* silver nanoparticles showed in Fig. 4. The analysis was evaluated and confirmed attachment of the functional group of the silver nanoparticles.

### X-Ray diffraction analysis (XRD) and dynamic light scattering (DLS)

**Figure 5:**
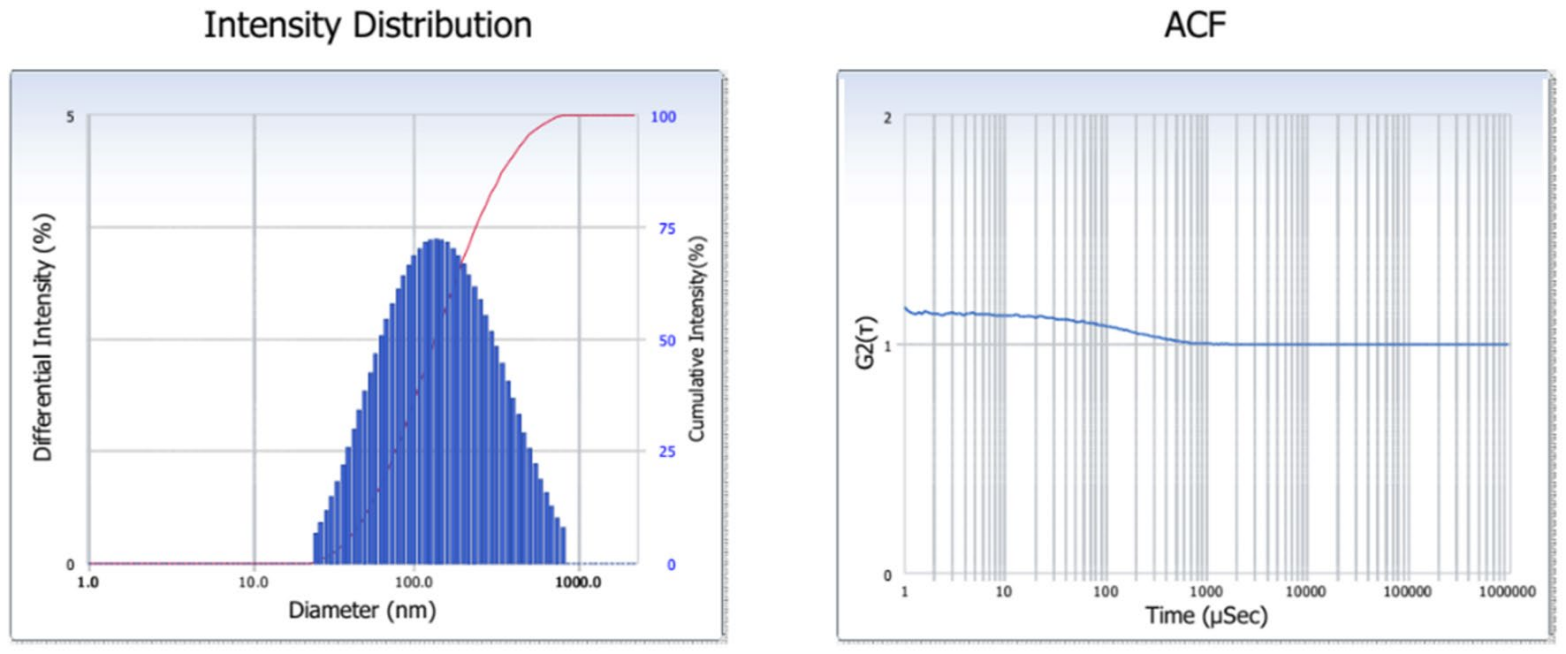
Analysis of Silver nanoparticles using DLS of Sargassum polycystum.

The crystallite nature of the synthesized silver nanoparticles was determined by XRD. Silver nanoparticles of *Sargassum polycystum* XRD showed in Fig. 4B. XRD peak values appeared at13.75, 27.45, 33.33,38.44. in the 2Ɵ range of 0° to 80° parallel to the characteristic’s diffraction of the (111), (200), (311) and (222). X-Ray diffraction showed that a silver nanoparticle of the *Sargassum polycystum* was crystalline in structure. Silver nanoparticle particle size distribution were determined using dynamic light scattering methods confirm the presence of silver nanoparticles. Silver nanoparticles average size as Fig. 5.

### Scanning electron microscope (SEM) and transmission electron microscopy (TEM) imaging analysis of the silver nanoparticles

**Figure 6:**
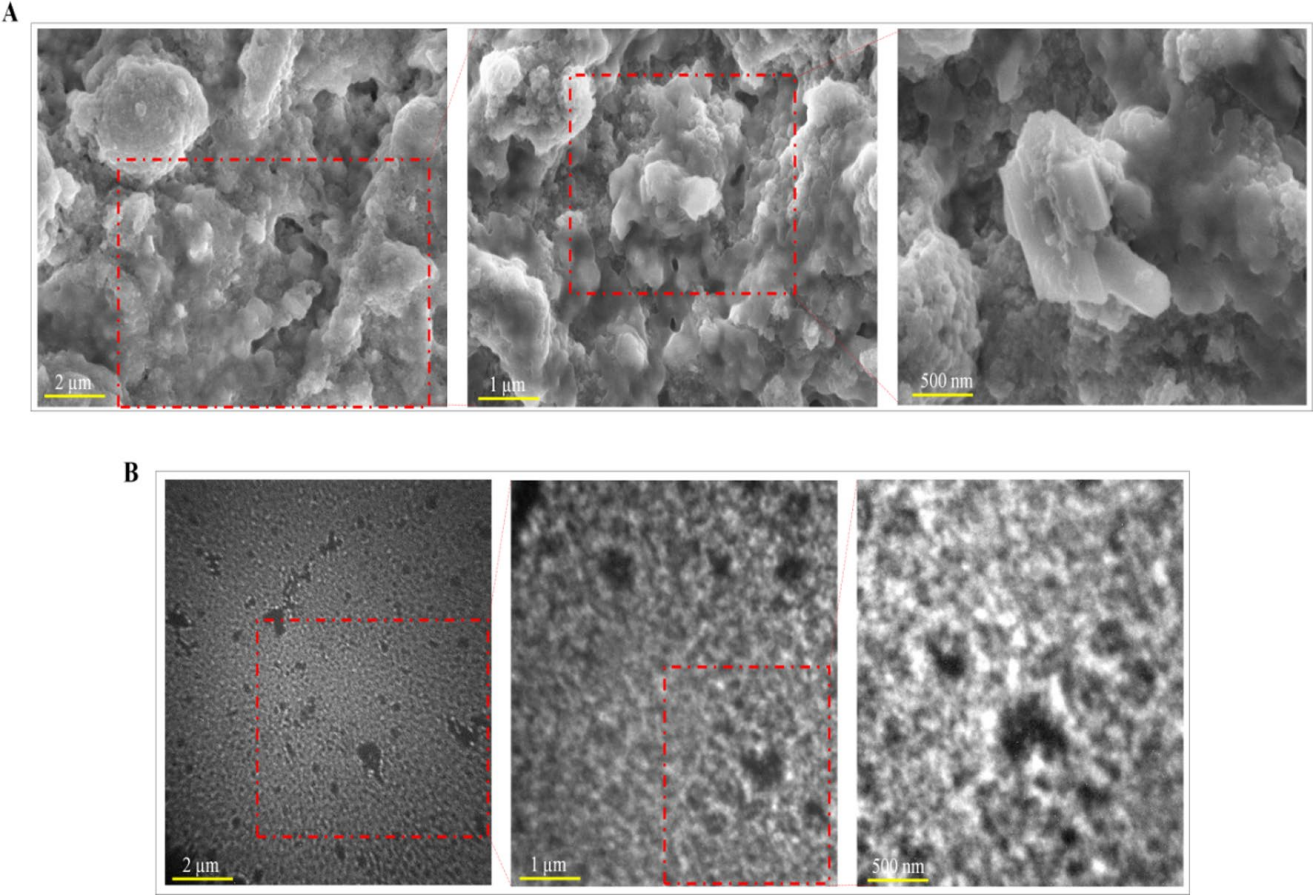
(**A**) Analysis of Silver nanoparticles using SEM of *Sargassum polycystum*. (**B**) Analysis of Silver nanoparticles using TEM of *Sargassum polycystum.*

SEM and TEM imaging analysis was performed synthesized *Sargassump olycystum* synthesized nanoparticles (Fig. 6A,B). It’s clearly indicated that nanoparticles are mostly cluster and spherical in shape and the size is less than 100 nm. Its denoted that formation of *S. polycystum* synthesized silver nanoparticles.

### Antibacterial and anti-mycobacterial activities of three different silver nanoparticles

**Figure 7:**
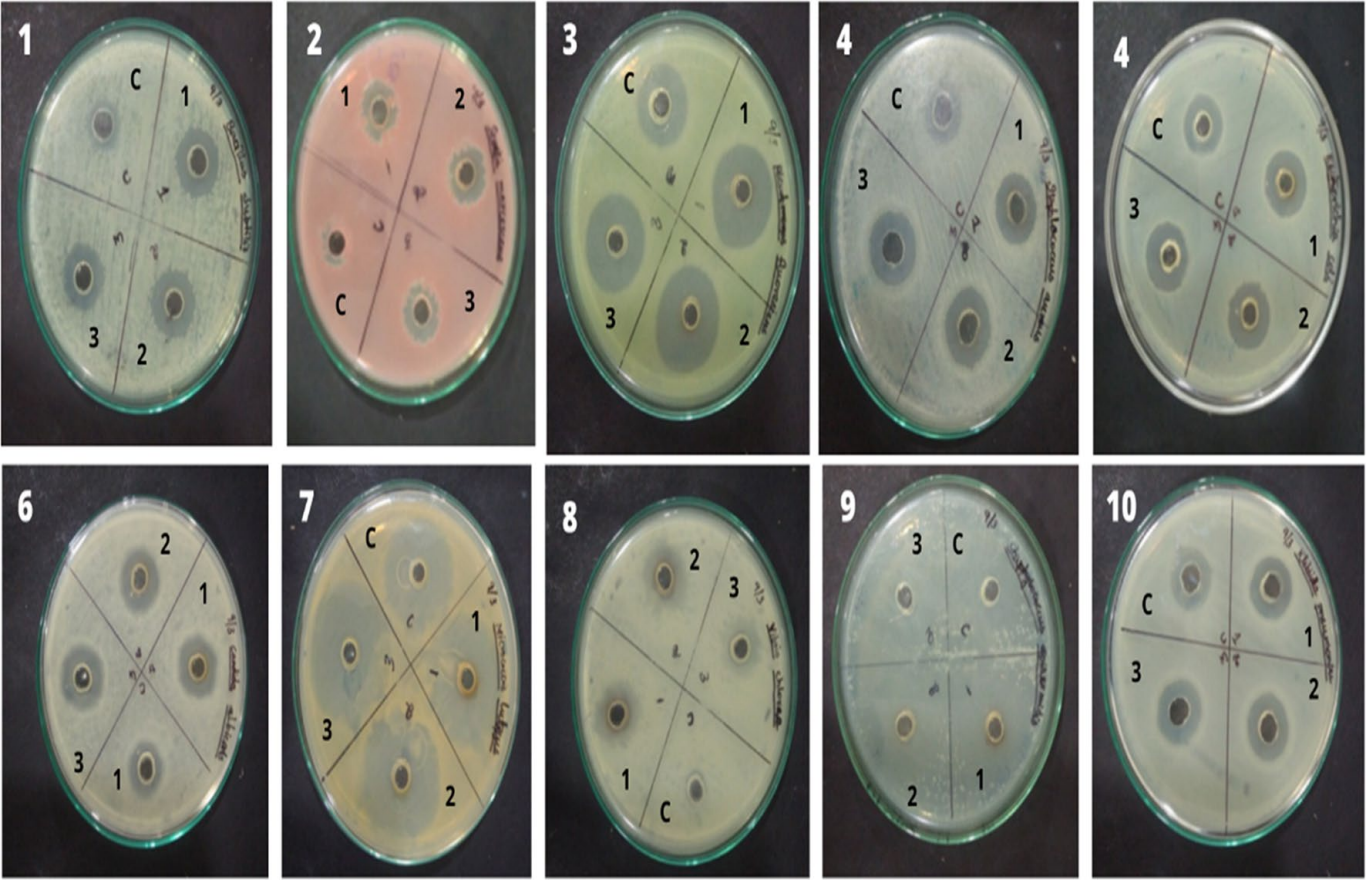
Antimicrobial activity of silver nanoparticles (1. *Sargassum polycystum,* 2 *Acanthophora spicifera, and* 3 *Ulva fasciata*). 1. *Bacillus subtilis,* 2*. Serratia marcescens,* 3*. Pseudomonas fluorescens,* 4*. Staphylococcus aureus,* 5*. Escherichia coli,* 6*. Candida albicans,* 7*. Micrococcus luteus,* 8*. Vibrio cholerae,* 9*. Staphylococcus epidermidis,* 10. *Klebsiella pnemoniae.*

*Sargassum polycystum* silver nanoparticles showed potential activities against *Staphylococcus aureus* showed a maximum 36 mm clear zone of inhibition, followed by *Micrococcus luteus *(35 mm), *Pseudomonas fluorescens* (25 mm), *Serratia marcescens*, *Klebsiella pnemoniae* and *Bacillus subtilis *(18 mm), *Escherichiacoli*, *Staphylococcus epidermidis* and *Candidaalbicans* (17 mm) and Vibrio cholera (15 mm) of inhibition in the antibacterial activities. *Acanthophoras picifera* nanoparticle showed maximum inhibition against *Staphylococcus aureus* (30 mm) followed by *Micrococcus luteus* (32 mm), *Pseudomonas fluorescens* (26 mm), *Bacillus subtilis* (18 mm), *Klebsiella pnemoniae*, *Escherichia coli*, *Staphylococcus epidermidis*, *Serratia marcescens* and *Vibrio cholera* (17 mm) and *Candida albicans *(15 mm). *Acanthophora spicifera* sliver nanoparticles showed maximum inhibition against *Micrococcus luteus* and *Staphylococcus aureus* (36 mm), followed by *Pseudomonas fluorescens* (24 mm), *Candida albicans* (20 mm), Escherichia *coli* (18 mm), *Serratia marcescens* (16 mm), *Klebsiella pnemoniae *(16 mm), *Bacillus subtili*s (17 mm), Staphylococcus *epidermidis *(17 mm) andminimum inhibition against Vibrio *cholera* (14 mm). *Sargassumwightii* silver nanoparticle showed maximum inhibition against *Micrococcus luteus* (30 mm) followed by *Staphylococcus aureus* (27 mm), *Pseudomonas fluorescens* (20 mm), *Klebsiella pnemoniae *(16 mm), *Escherichia coli* and *Candida albicans* (15 mm), *Staphylococcus epidermidis* (14 mm), *Bacillus subtilis* and minimum inhibition found in *Serratiamarcescens *(12 mm) and there is no inhibition found in *Vibrio cholera* (Fig. 7).

### Minimum inhibition concentration (MIC)

Based on the antibacterial activities *Bacillus subtilis*, *Micrococcus luteus*, *Staphylococcus aureus*, and *Serratiamarcescens* were chosen for the minimum inhibition assay. *Sargassum polycystum* silver nanoparticles showed maximum 47% of inhibition found at 16 µg/ml against *Escherichia coli*, followed by *Micrococcus luteus* 21% of inhibition, *Serratiamarcescens* 12% and *Bacillus cereus* 11% inhibition. *Sargassum polycystum* silver nanoparticle showed 15% inhibition against *Candida albicans* at 32 µg/ml.

### Anti-mycobacterial activities

**Figure 8:**
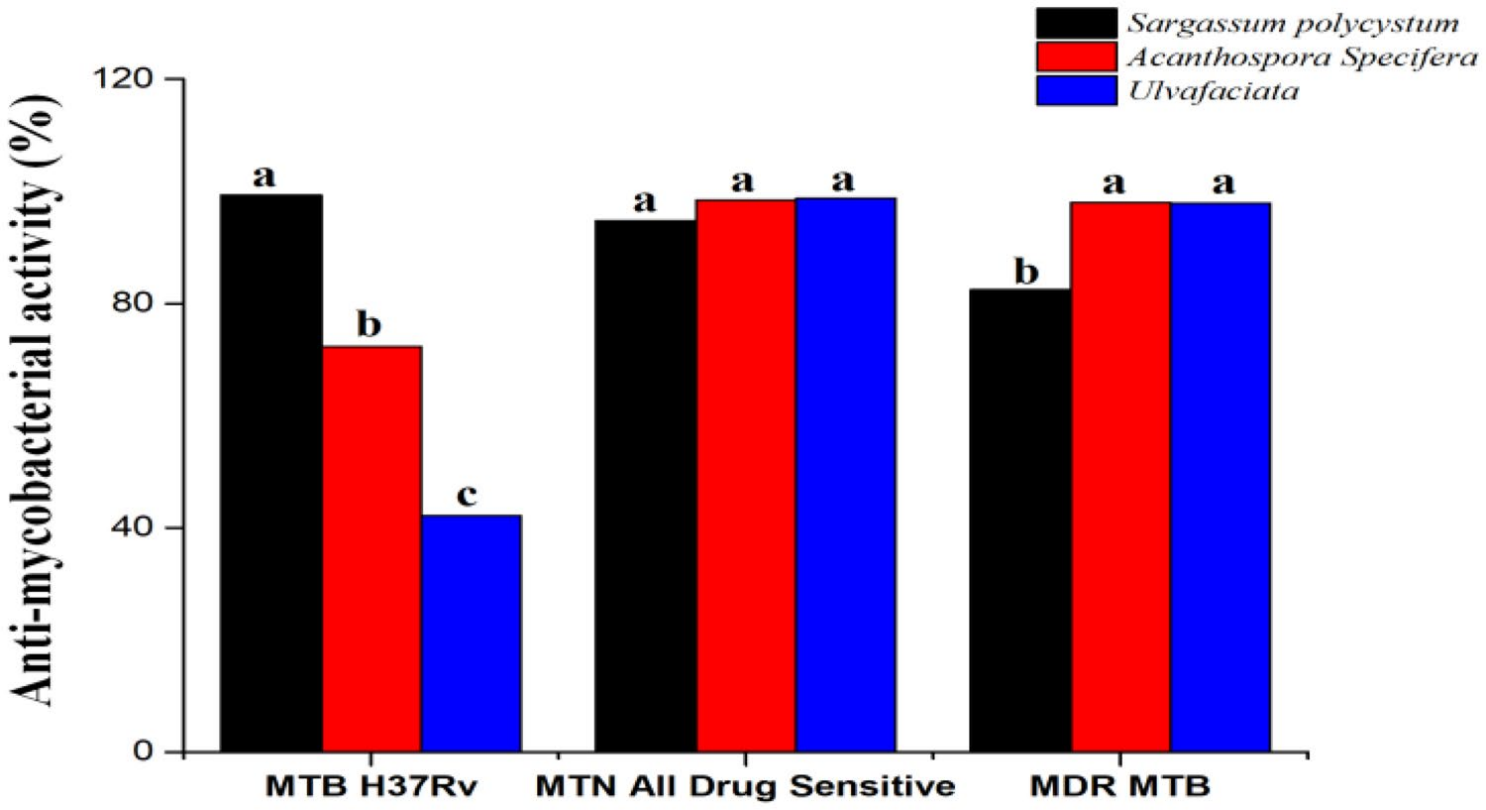
Anti-Mycobacterial activity against silver nanoparticle Seaweeds.

The anti-mycobacterial activity of *Sargassum polycystum* showed prospective activities against *mycobacterium tuberculosis*. *S. polycystum* showed 99.38%, 94.79% and 82.44% of inhibition against MTBH37Rv, MTB all drug sensitive and MTD MTB respectively. *Acanthophora spicifera* showed 72.31%, 98.42%and 97.96% inhibition against MTBH37Rv, MTBalldrug sensitive and MTD MTB respectively. *Sargassum wightii* showed 42.17%, 98.75% and 97.89% inhibition against MTBH37Rv, MTB all drug sensitive and MTD MTB respectively (Fig. 8).

### Toxicity evaluation of silver nanoparticles

**Figure 9:**
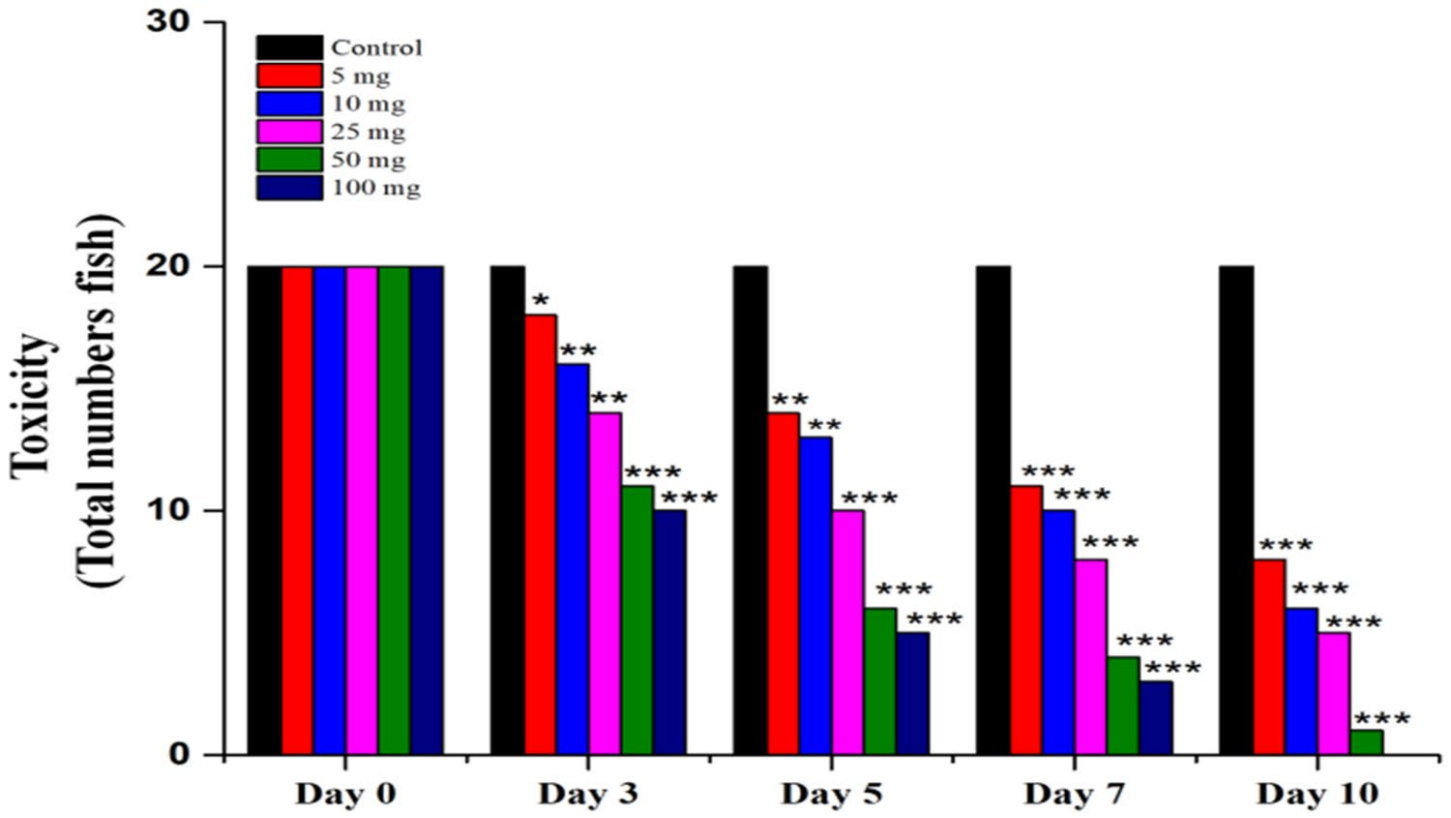
Toxicity study of silver nanoparticles of *Sargassum polycystum* zebra fish embryo.

We have used five different concentrations of silver nanoparticles ranging from 5, 10, 25, 50 and 100 mg were used for evaluate the toxicity of nanoparticles. There is no significant mortality was observed at 5 mg of the Silver nano particles of *Sargassum polycystum* showed a less toxicity in zebra fish larvae (Fig. 9).

## Discussion

The chemical reduction process has been used to create silver nanoparticles with success. Visual observation of the production of silver nanoparticles revealed colouring (yellowish). The sample's development of a yellowish colour is an indication that silver nanoparticle particlesdominated the synthesis process, which resulted in colloidal nanoparticles^17^. In our present studies also showed that the three seaweeds extract showed a colour change after mixing mixed with silver nitrate. After incubation colour change was observed. The confirmation in the experiments was of the formation Yellowish-brown colours. There was no colour change in control silver nitrate solution. Silver nanoparticles were confirmed by using UV-spectrophotometer.

It was noted that the colour of the solution changed from colourless to yellowish-brown with the addition of marine macroalgae extracts, indicating the biogenesis of AgNPs. The aqueous medium's obvious colorlessness clearly suggests that extracellular reduction of Ag^+^ ions had not taken place. It is widely known that the generation of smaller-sized NPs and reduction processes both depend greatly on the alkaline pH^18^. Secondary confirmations of the formation of the silver nanoparticles were determined by UV–VIS spectrophotometer. It showed single strong absorption at 422,429 and 411 nm in UV–Vis spectrum of *Sargassum polycystum*, *Acanthophora spicifera*, and *Sargassum wightii* respectively. This absorption band called as surface plasmon resonance (SRP). UV–Vis spectra of AgNPs formed by the extracts of different algalspecies. Absorption peaks of AgNPs capped by *U. rigida*, *C. myrica*, and *G. foliifera* appearedat 424 nm, 409 nm, and 415 nm, respectively. These types of UV–VIS spectroscopy absorption peak obtained by Algotiml et al.^19^. They reported that silver nanoparticles showed a peak value at 422 and 425 nm for *Codiumcapitatum* seaweeds of dried and fresh respectively. This type of surface Plasmon vibration with characteristic peaks of silver nanoparticles was prepared by chemical reduction^20^.

The last ten years have seen a surge in interest in the field of catalytic processing of algal biomass. Future economic growth from algae should be significant if efficient upstream and downstream processing can be created. Algae are well known for their ability to transform into more pliable forms and to hyper-accumulate heavy metal ions^21^. Due to their ability to reduce metal ions, the creation of nanoparticles from a variety of algal sources has emerged as one of the most cutting-edge and current fields of biochemical study^22^. In the realm of materials science, green synthesis has emerged as a dependable, long-lasting, and environmentally friendly method for the synthesis of several nanomaterials, including metal oxides, hybrids, and bio-inspired materials^23^. Metallic nanoparticles have fascinated scientists for more than a century and are now widely used in engineering and the health sciences^24^.

Sivakumar et al. reported the silver nanoparticles from seaweed samples were found to have antibacterial activity. The zone of inhibition in the plate demonstrated that silver nanoparticles made from seaweed samples. *Staphylococcus aureus* was shown to be more vulnerable to silver nanoparticles than gram negative bacteria by comparing the zones of inhibition between these bacteria (*Salmonella typhi*). Compared to those from *T. conoides* and *H. macroloba*, silver nanoparticles from *S. filamentosa* had a noticeably higher activity against *Staphylococcusaureus*. Silver nanoparticles from *T. conoides* and *S. filamentosa* have much stronger anti-*Salmonella* activity than those from *H. macroloba*^25^. In our present studies also supported that silver nanoparticles from marine seaweeds showed the potential activities human pathogens.

Fourier transforms infrared spectroscopy analysis of *Sargassum polycystum* silver nanoparticles showed in the Table 1. Fourier transforms infrared spectroscopy analysis evaluated the functional group of the silver nanoparticles. Fourier transforms infrared spectroscopy (FTIR) spectrum was used to evaluate the functional or bio molecules in green synthesis of silver nanoparticles using *Sargassum polycystum*. The peaks 3888.62 cm^−1^ indicating the presence of Alcohol (O–H), than 3395.99 (cm^−1^) indicating the presence of Stretching of Amide II (N–NH), followed by 2921.93 (cm^−1^) indicating the Aklanes (–CH2–), 2129.99 (cm^−1^) demonstrating Alkyanes (–C–C)group, band was absorbed at 1582.96 (cm^−1^), is due to the presence of aromatic ring (C=C) Most of the marine seaweeds contain high amount of phenolic and flavonoids. This type of similar type of compounds reported in the seaweeds of *Caluerpataxifolia* silver nanoparticles^26^.

**Table 1:** Fourier transforms infrared spectroscopy analysis.

Peak values	Functional groups
3888.62 (cm^**−1**^)	Alcohol (O–H),
3395.99(cm^**−1**^)	Amide II (N–NH)
2921.93 (cm^**−1**^)	Aklanes (–CH2–)
1582.96 (cm^**−1**^)	Aromatic ring (C=C)

Then XRD used to confirm the particles as silver and know the structural information. X-ray diffraction was one of the important characterizes the silver nanoparticles of *Sargassum polycystum*. Silver nanoparticles crystallite nature determined by XRD. XRD peak values appeared at 13.75, 27.45, 33.33, 38.44. in the 2Ɵ range of 0° to 80° parallel to the characteristics diffraction of the (111), (200), (311) and (222). X-Ray diffraction showed that a silver nanoparticle of the *Sargassum polycystum* was crystalline in structure.

Sivakumar et al.^26^ reported that silver nanoparticles of the *Halimeda macroloba*, *Turbinaria conoides* and *Spyridia filamentosa*. XRD spectrum may be used to determine the precise nature of the silver particles that were produced. The broadness of the X-ray diffraction peaks at their bottoms suggests that the silver particles are nanoscale. The X-ray diffraction lines' peak spreading at half their maximum intensity is caused by crystallite size, flattening and micro-strainsinside the diffracting domains. The cubic silver's crystalline planes' unique diffraction peaks were revealed by XRD investigation (Ag). There were peaks in all three samples, which were noted at 111, 200, 220 and 311. It can be denoted that for the four samples, the values of were 28°, 32°, 41.5° and 49.5°. Analysis of Silver nanoparticles using SEM result showed that silver nanoparticles are less than 100 nm in size. Average diameter of the silver nanoparticles was between 10 and 85 nm in size. TEM analysis of the silver nanoparticles showed that *Sargassum polycystum* are mostly spherical in shape.

Researchers mostlyuse the TEM and SEM to show how different sizes of nanoparticles evolve. In order to reduce charging artifacts and quick radiation damage to biomaterials during the imaging process, SEM imaging requires a thorough preparation step that is frequently finished by metal coating. Additionally, for improved identification, SEM and TEM are combined with an electron diffraction (EDX) equipment. In order to prepare samples, carbon copper grids are coated with a metal nanoparticle solution, dried, and then ready for measurement. The crystalline structure of nano-metals will be revealed via an X-ray diffraction (XRD) investigation.

EDS (energy-dispersive spectroscopy) is another tool that is frequently used to detect the presence of metal^27^. EDS systems are often incorporated with SEM or EPMA equipment. An EDS system consists of a sensitive x-ray detector, a liquid nitrogen dewar for cooling, and software for gathering and analysing energy spectra. These techniques make it simple for researchers to quantify the structural properties of nanomaterials.

We used 5 different concentrations of silver nanoparticles such as 5 mg, 10 mg, 25 mg, 50 mg and 100 mg used for the toxicity studies. In these studies, 5 mg of the Silver nanoparticles of *Sargassum polycystum* showed a less toxicity in zebra fish larvae (Supplementary Table S1).

## Conclusion

Green synthesis of macro algae coated silver particle showed significant antibacterial and anti-mycobacterial activity. Among three different types seaweeds *Sargassum polycystum*coated synthesized silver nanoparticles showed the significant activity against pathogens . The silver nanoparticles were characteristic with various spectroscopic analyses. There is no or less toxicity was observed against Zebra fish. It could act as alternative agent for antibacterial and anti-mycobacterial activity.

## Supplementary Information

Supplementary Tables.

## Data Availability

All the images given in the article are obtained based on experimental data. None of the images were reproduced from other sources. The datasets used and/or analysed during the current study available from the corresponding author on request.
