# Author Correction: Silver nanoparticles synthesized from the seaweed *Sargassum polycystum* and screening for their biological potential

**DOI:** 10.1038/s41598-022-22983-7

**Published:** 2022-11-01

**Authors:** Rajasekar Thiurunavukkarau, Sabarika Shanmugam, Kumaran Subramanian, Priyadarshini Pandi, Gangatharan Muralitharan, Maryshamya Arokiarajan, Karthika Kasinathan, Anbarasu Sivaraj, Revathy Kalyanasundaram, Suliman Yousef AlOmar, Velmurugan Shanmugam

**Affiliations:** 1 Centre for Drug Discovery and Development, Col. Dr. Jeppiaar Research Park, Sathyabama Institute of Science and Technology Jeppiaar Nagar, Rajiv Gandhi Road, Chennai, 600 119 India; 2 Departmentof Microbiology, Centre of Excellence in Life Sciences, Bharathidasan University, Tiruchirappalli, 620 024 Tami Nadu India; 3 Department of Biotechnology, Mohamed Sathak College of Arts and Science, Chennai, Tamil Nadu India; 4 Department of Zoology, College of Science, Kind Saud University, Riyadh, 11451 Kingdom of Saudi Arabia; 5 Madda Walabu University, Robe, Ethiopia

Correction to: *Scientific Reports* 10.1038/s41598-022-18379-2, published online 30 August 2022

The Funding section in the original version of this Article was omitted. The Funding section now reads:

“This project was supported by the Researchers Supporting Project number (RSP-2021/35), King Saud University, Riyadh, Saudi Arabia”.

The original Article has been corrected.

