# Supplementary material for: Silver nanoparticles synthesized from the seaweed Sargassum polycystum and screening for their biological potential
Source: Sci Rep. 2022 Aug 30;12:14757. doi: 10.1038/s41598-022-18379-2 (PMC9427740; doi:10.1038/s41598-022-18379-2)
Supplement: Supplementary file 1 — Supplementary Tables. [file 41598_2022_18379_MOESM1_ESM.docx]

Table: **Phytochemical content of seaweeds in three different solvents** (+) indicates presence; (- )indicates absence

| Phytochemical  parameters | *Sargassum polycystum* | *Sargassum wightii* | *Acanthophora spicifera* | |
| --- | --- | --- | --- | --- |
|  | Ethanol | Ethanol | Ethanol |  |
| Alkaloids | **-** | **+** | **++** |  |
| Terpenoids | **+** | **-** | **-** |  |
| Steroids | **-** | **-** | **-** |  |
| Tannins | **+** | **+** | **+** |  |
| Saponins | **-** | **++** | **-** |  |
| Flavonoids | **++** | **-** | **++** |  |
| Phenols | **-** | **-** | **+** |  |
| Coumarins | **+** | **+** | **-** |  |
| Quinones | **-** | **-** | **-** |  |
| Glycosides | **-** | **-** | **-** |  |

Table 1: Fourier transforms infrared spectroscopy analysis

| Peak Values | Functional groups |
| --- | --- |
| 3888.62 (cm**^-1^**) | Alcohol (O-H), |
| 3395.99(cm**^-1^**) | Amide II (N-NH), |
| 2921.93 (cm**^-1^**) | Aklanes (-CH2-), |
| 1582.96 (cm**^-1^**) | aromatic ring (C=C) |
